# Impact of a Blended Periconception Lifestyle Care Approach on Lifestyle Behaviors: Before-and-After Study

**DOI:** 10.2196/19378

**Published:** 2020-09-30

**Authors:** Melissa van der Windt, Rianne Maria van der Kleij, Katinka Marianne Snoek, Sten Paul Willemsen, Ramon Henny Maria Dykgraaf, Joop Stephanus Elisabeth Laven, Sam Schoenmakers, Régine Patricia Maria Steegers-Theunissen

**Affiliations:** 1 Department of Obstetrics and Gynecology Erasmus University Medical Center Rotterdam Netherlands; 2 Public Health and Primary Care Leiden University Medical Center Leiden Netherlands; 3 Department of Biostatistics Erasmus University Medical Center Rotterdam Netherlands

**Keywords:** eHealth, periconception period, lifestyle intervention

## Abstract

**Background:**

Periconception lifestyle behaviors affect maternal, paternal, offspring, and transgenerational health outcomes. Previous research in other target populations has shown that personalized lifestyle interventions, in which face-to-face counseling and eHealth (“blended care”) are combined, may effectively target these lifestyle behaviors.

**Objective:**

We aimed to assess the effectiveness of a periconceptional lifestyle intervention on the improvement of specific lifestyle components.

**Methods:**

A blended periconception lifestyle care approach was developed, combining the outpatient lifestyle counseling service “Healthy Pregnancy” with the eHealth platform “Smarter Pregnancy” (www.smarterpregnancy.co.uk) in which lifestyle was coached for 24 weeks. All couples contemplating pregnancy or already pregnant (≤12 weeks of gestation) who visited the outpatient clinics of the Department of Obstetrics and Gynecology at the Erasmus University Medical Center (Erasmus MC), Rotterdam, the Netherlands, between June and December 2018, were invited to participate. We measured changes in lifestyle behaviors at weeks 12 and 24 compared with baseline. Generalized estimating equations were used to analyze the changes in lifestyle behaviors over time. Subgroup analyses were performed for women with obesity (BMI ≥30 kg/m^2^), women pregnant at the start of the intervention, and those participating as a couple.

**Results:**

A total of 539 women were screened for eligibility, and 450 women and 61 men received the blended periconception intervention. Among the participating women, 58.4% (263/450) were included in the preconception period. Moreover, 78.9% (403/511) of the included participants completed the online lifestyle coaching. At baseline, at least one poor lifestyle behavior was present in most women (379/450, 84.2%) and men (58/61, 95.1%). In the total group, median fruit intake increased from 1.8 to 2.2 pieces/day (*P*<.001) and median vegetable intake increased from 151 to 165 grams/day (*P*<.001) after 24 weeks of online coaching. The probability of taking folic acid supplementation among women increased from 0.97 to 1 (*P*<.001), and the probability of consuming alcohol and using tobacco in the total group decreased from 0.25 to 0.19 (*P*=.002) and from 0.20 to 0.15 (*P*=.63), respectively. Overall, the program showed the strongest effectiveness for participating couples. Particularly for vegetable and fruit intake, their consumption increased from 158 grams/day and 1.8 pieces/day at baseline to 190 grams/day and 2.7 pieces/day at the end of the intervention, respectively.

**Conclusions:**

We succeeded in including most participating women in the preconception period. A high compliance rate was achieved and users demonstrated improvements in several lifestyle components. The blended periconception lifestyle care approach seems to be an effective method to improve lifestyle behaviors. The next step is to further disseminate this approach and to perform a randomized trial to compare the use of blended care with the provision of only eHealth. Additionally, the clinical relevance of these results will need to be substantiated further.

## Introduction

A healthy lifestyle helps to optimize the well-being and health outcomes of individuals across their life course. Previous research has shown that adhering to a healthy lifestyle is associated with an increase in the quality of life and life expectancy [1-5]. The exact definition of a “healthy lifestyle” varies over time and between authors. Predominantly, a “healthy lifestyle” is referred to as a combination of lifestyle components associated with lower mortality and better health outcomes. Most definitions include components such as nutrition, physical activity, no smoking, and minimal alcohol consumption [6-10]. According to the World Health Organization, healthy nutrition can be further specified as the consumption of at least 400 grams of fruits and vegetables per day, less than 5 mg of salt per day, and limited consumption of (free) sugars (maximum of 25 gram per day), saturated fats (less than 10% of total energy intake), and trans-fats (less than 1% of total energy intake) [11].

The impact of parental lifestyle around the time of conception on maternal, paternal, fetal, and neonatal health is a focus of increasing interest. The periconception period commences 14 weeks before conception and ends 10 weeks after conception. During this period, adherence to a healthy lifestyle is critical for optimizing gamete function, early placentation, and embryonic development [12]. These processes influence both offspring health and future health across generations [13]. Therefore, the periconception period is considered the “window of opportunity” in which the foundation for optimum growth, development, and health across the life course is established.

A lifestyle intervention can be defined as a behavioral intervention method to improve an individual’s lifestyle by addressing the optimization of one or more lifestyle components. A lifestyle intervention during the periconception period has the potential to effectively target unhealthy lifestyle components and thereby improve reproductive outcome and the future health of both parents and their offspring [12]. However, several interventions have aimed to influence maternal lifestyle, but the time window to do so has been mostly too short to make an impact, with interventions starting when pregnancy is well underway [14]. Moreover, most interventions were not personalized to the individual couple, making it difficult to address their individual needs and wants. A lifestyle intervention that starts early, preferably in the preconception period, takes couples into account (not only women), and integrates the foundations of personalized medicine is therefore warranted to address the lifestyle of women and men throughout the pregnancy and perinatal periods [14-16].

The Erasmus University Medical Center (Erasmus MC) developed and tested the outpatient lifestyle counseling clinic “Achieving a Healthy Pregnancy,” and this intervention was demonstrated to decrease the prevalence of unhealthy lifestyle components in a sample of 419 mostly subfertile couples [17]. Additionally, the Erasmus MC developed and evaluated the eHealth coaching program “Smarter Pregnancy” in a large survey including both a general population cohort and a subfertile (in vitro fertilization/intracytoplasmic sperm injection) cohort [18-20].

Interventions that combine face-to-face counseling with eHealth (referred to as “blended care”) are increasingly applied in the context of mental health care [21]. Moreover, promising results have been shown for using blended care in lifestyle medicine [22]. With blended care, intervention effectiveness may be enhanced, as this treatment modality improves user motivation, engagement, and self-management, and decreases intervention resistance (and dropouts) and the number of hospital consultations. As such, blended care has the potential to reduce total treatment cost [23,24]. However, its benefits have mainly been proven in mental health care and have not been confirmed in the specific setting of periconception lifestyle care. We therefore developed and evaluated a blended personalized lifestyle care approach to periconception care, combining an outpatient lifestyle counseling service with the eHealth platform “Smarter Pregnancy” [25]. We aimed to assess the effectiveness of the lifestyle intervention on the improvement of the specific lifestyle components vegetable and fruit intake, folic acid supplement use, tobacco use, and alcohol consumption. Furthermore, intervention compliance rates were determined. Subgroup analyses were performed to evaluate the intervention effectiveness for specific subgroups, such as pregnant women, women whose partners also participated, women suffering from obesity, and men.

## Methods

### Study Design and Participants

All couples contemplating pregnancy or those already pregnant (≤12 weeks of gestation) who visited the outpatient clinics of the Department of Obstetrics and Gynecology at the Erasmus MC, Rotterdam, the Netherlands, were invited to participate. In our study, we invited couples to participate between June 2018 and December 2018. The exclusion criteria were pre-existing type 1 diabetes mellitus, insufficient knowledge of the Dutch language, and inability to provide informed consent. A woman could be included as a single participant if her partner did not participate.

This study was approved by the medical ethics and institutional review board of the Erasmus MC, Rotterdam, the Netherlands (MEC-2018-1232). Written informed consent was obtained from all female and male participants at enrollment.

### Intervention

**Figure 1:**
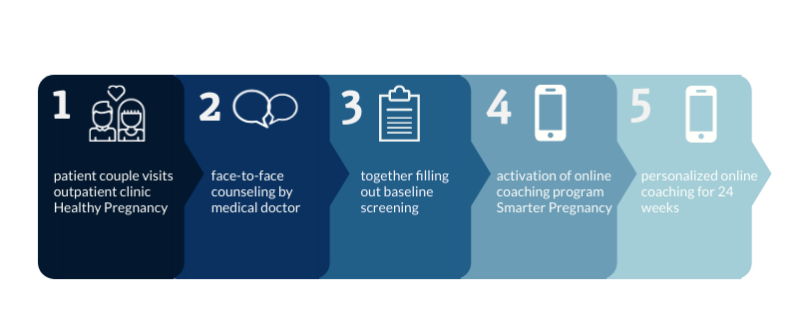
Diagram of the study process.

The blended care approach comprised the following two integrated parts: the outpatient clinic Healthy Pregnancy and the online eHealth coaching program Smarter Pregnancy. The study process is presented in Figure 1. The outpatient clinic Healthy Pregnancy was based on the previously proven to be effective outpatient clinic Achieving a Healthy Pregnancy [17]. The online eHealth program included 6 months of personalized coaching on the most prevalent inadequate nutrition and lifestyle behaviors (vegetable, fruit, and folic acid intake, tobacco use, and alcohol consumption). Use of the online coaching platform improved lifestyle components of the participants after 6 months by 26.3% (95% CI 23.0-29.9) for vegetable intake, 38.4% (95% CI 34.5-42.5) for fruit intake, 56.3% (95% CI 48.8-63.6) for folic acid supplement use, 35.1% (95% CI 29.1-41.6) for no tobacco use, and 41.9% (95% CI 35.2-48.9) for no alcohol consumption.

The counselor, a medical doctor trained in motivational interviewing, filled out the baseline screening of Smarter Pregnancy [25] together with the couple during a visit to the outpatient clinic Healthy Pregnancy. Following the results of the baseline screening of Smarter Pregnancy, the counselor provided the couple with tailored lifestyle advice and possible options to alter their lifestyle. The information obtained during the face-to-face session was used to personalize the 6 months of online coaching provided by the eHealth platform Smarter Pregnancy. Participating couples, both women and men, received up to three short motivating and supporting messages per week by email. These messages included vouchers, seasonal recipes, and personalized tips and recommendations to achieve a healthy diet and to increase physical activity. Every 6 weeks, additional questions addressing behavior, diet, and pregnancy status were sent to monitor lifestyle changes. All participants (both women and men) had access to their personal page on the online Smarter Pregnancy platform, which provided additional modules to encourage the performance of physical activity, increase compliance with hospital appointments, and optimize medication adherence. A summary of all individual results can also be extracted or shared with the health care professional for further evaluation and support of preconception and antenatal care.

### Measurement Instruments and Data Collection

The lifestyle indicators weight and height were measured during the face-to-face appointment. The BMI was calculated by dividing weight in kilograms by the square of height in meters. Using a lifestyle questionnaire integrated in the online coaching program Smarter Pregnancy, baseline screening and follow-up screening at weeks 6, 12, 18, and 24 of the program were performed. The follow-up screening was used to monitor the change in lifestyle components and the pregnancy state. At weeks 12 and 24 of the program, participants were invited to fill out a short questionnaire about all lifestyle components and the pregnancy state. At weeks 6 and 18, they received a short questionnaire that only included questions about lifestyle components that were found to be inadequate at baseline. The lifestyle questionnaire included questions regarding vegetable and fruit intake, folic acid supplement use, tobacco use, and alcohol consumption.

### Outcomes

The blended care approach focused on the following five lifestyle components: vegetable and fruit intake, folic acid supplement use, tobacco use, and alcohol consumption. Adequate lifestyle behaviors, considered as a healthy lifestyle, were formulated as intake of at least 200 grams of vegetables per day, intake of at least two pieces of fruit per day, low dose (400 µg) folic acid supplement use (only for female participants), no tobacco use, and no alcohol consumption.

### Statistical Analysis

Participants who completed the blended lifestyle care approach and those who resigned prematurely were included in the analysis. Compliance was defined as the percentage of participants who completed the 6-month blended care approach. Baseline characteristics of the study participants were computed as medians or percentages and were calculated separately for women who were pregnant at the start of the approach, women who were trying to conceive, and male participants. Vegetable intake and fruit intake were analyzed as continuous variables. The results of dichotomous variables, folic acid supplement use, tobacco use, and alcohol consumption were shown as probabilities.

Generalized estimating equations were used to analyze the changes in lifestyle behaviors. Subgroup analyses were performed for obese women (BMI ≥30 kg/m^2^), pregnant women at the start of the program, and couples. We hypothesized that women with obesity may have more unhealthy lifestyle behaviors, pregnant women may have healthier lifestyle behaviors, and women who participated with their partners may be more responsive to the blended care approach. A *P* value <.05 was considered statistically significant. All analyses were performed using SPSS package 21.0 (IBM SPSS Statistics) and R (R for Windows, version 3.5; R Core Team).

## Results

### Study Population

A total of 511 patients (450 women and 61 men) received the blended lifestyle care approach. Additionally, 58.4% (263/450) of female participants were included during the preconception period. The median BMI among female participants was 24.8 kg/m^2^. Among female participants, 27.6% (124/450) were overweight (BMI 25-30 kg/m^2^) and 20.9% (94/450) were obese (BMI ≥30 kg/m^2^). The median BMI among male participants was 25.5 kg/m^2^. Among male participants, 38.2% (21/55) were overweight and 16.4% (9/55) were obese. The intervention compliance rate was 78.9% (403/511) among all participants. The highest compliance, defined as the percentage of participants who completed the online coaching program Smarter Pregnancy, was achieved in the group of women who were not pregnant at the start of the approach (215/263, 81.7%).

### Baseline Lifestyle Behaviors

The median vegetable intake was 157 grams per day for women and 146 grams per day for men (Table 1). Adequate vegetable intake was achieved in 31.6% (137/434) of participating women and 25.0% (14/56) of participating men. The median fruit intake was 1.9 pieces per day for women and 1.4 pieces per day for men. Adequate fruit intake was observed in 49.1% (212/432) and 27.3% (15/55) of women and men, respectively. Folic acid intake was adequate at baseline in 76.7% (345/450) of participating women, and was adequate in 96.8% (181/187) and 62.4% (164/263) of women pregnant at the start of the program and nonpregnant women, respectively. Regular tobacco use was reported by 8.8% (38/431) of all women and 4.6% (8/175) of women who were pregnant at the start of the approach. On the other hand, 20.0% (11/55) of men reported tobacco use, and their partners were predominantly in the preconception period (10/11, 90.9%). Alcohol consumption was reported by 20.5% (88/430) of participating women, 1.1% (2/175) of women who were pregnant at the start of the approach, and 65.5% (36/55) of men. All baseline lifestyle behaviors were worse in nonpregnant women and men compared with women who were pregnant at the start of the approach. Subgroup analyses of lifestyle behaviors between normal weight (n=356) and obese women (n=94) showed no relevant differences at baseline (Table 2).

**Table 1:** Baseline characteristics of the study participants stratified by sex and pregnancy status.

Characteristic		Women (N=450)	Pregnant women (n=187)	Nonpregnant women (n=263)	Men (N=61)
**Age**					
	Overall value (years), median (IQR)	32.3 (28.5-36.2)	31.8 (28.0-35.2)	32.5 (29.0-36.6)	33.6 (30.9-39.5)
	Missing data, n	0	0	0	0
**BMI**					
	Overall value (kg/m^2^), median (IQR)	24.8 (22.1-29.1)	24.5 (21.9-29.0)	25.2 (22.3-29.4)	25.5 (22.6-29.0)
	Overweight (BMI 25-30 kg/m^2^), median (IQR)	27.5 (25.8-28.7)	27.3 (25.7-28.8)	27.5 (26.0-28.7)	27.7 (25.9-29.0)
	Overweight, n (%)	124 (27.6%)	46 (24.6%)	78 (29.7%)	21 (38.2%)
	Obese (BMI 30-60 kg/m^2^), median (IQR)	32.9 (31.5-35.6)	32.0 (31.6-35.9)	33.8 (31.6-35.9)	34.5 (31.2-38.7)
	Obese, n (%)	94 (20.9%)	37 (19.8%)	57 (21.7%)	9 (16.4%)
	Missing data, n	0	0	0	6
**Adequate folic acid intake**					
	Value, n (%)	345 (76.7%)	181 (96.8%)	164 (62.4%)	N/A^a^
	Missing data, n	0	0	0	N/A
**Vegetable intake**					
	Overall value (grams/day), median (IQR)	157 (100-214)	157 (100-207)	150 (100-214)	146 (93-209)
	Adequate (≥200 grams/day), n (%)	137 (31.6%)	51 (29.0%)	86 (33.3%)	14 (25.0%)
	Missing data, n	16	11	5	5
**Fruit intake**					
	Overall value (pieces/day), median (IQR)	1.90 (0.94-3.11)	2.3 (1.3-3.7)	1.7 (0.8-2.5)	1.4 (0.8-2.1)
	Adequate (≥2 pieces/day), n (%)	212 (49.1%)	106 (60.6%)	106 (41.2%)	15 (27.3%)
	Missing data, n	18	12	6	6
**Smoking**					
	Smoking (yes), n (%)	38 (8.8%)	8 (4.6%)	30 (11.7%)	11 (20.0%)
	Missing data, n	19	12	7	6
**Alcohol use**					
	Alcohol use (yes), n (%)	88 (20.5%)	2 (1.1%)	85 (33.3%)	36 (65.5%)
	Missing data, n	20	12	8	6
Completed the program (yes), n (%)		358 (79.6%)	143 (76.5%)	215 (81.7%)	45 (73.8%)

^a^N/A: not applicable.

**Table 2:** Differences in lifestyle behaviors at baseline between normal weight and overweight women and obese women.

Characteristic		Women (N=450)		*P* value
		Normal weight and overweight (n=356)	Obese (n=94)	
**Age**				.20
	Overall value (years), median (IQR)	32.5 (28.6-36.5)	31.6 (28.4-34.5)	
	Missing data, n	0	0	
**Pregnancy status**				.63
	Pregnant (yes), n (%)	150 (42.1%)	37 (39.4%)	
	Missing data, n	0	0	
**BMI**				<.001
	Overall value (kg/m^2^), median (IQR)	23.8 (21.5-26.0)	32.8 (31.5-35.6)	
	Missing data, n	0	0	
**Adequate folic acid intake**				.10
	Value (no), n (%)	84 (23.9%)	36 (38.3%)	
	Missing data, n	0	0	
**Vegetable intake**				.13
	Overall value (grams/day), median (IQR)	157 (100-214)	128 (93-214)	
	Missing data, n	13	3	
**Fruit intake**				.07
	Overall value (pieces/day), median (IQR)	2.08 (0.95-3.24)	1.6 (0.7-2.5)	
	Missing data, n	15	3	
**Smoking**				.12
	Smoking (yes), n (%)	27 (7.6%)	12 (13.2%)	
	Missing data, n	16	3	
**Alcohol use**				.34
	Alcohol use (yes), n (%)	72 (20.2%)	15 (16.7%)	
	Missing data, n	16	4	

### Effectiveness

**Figure 2:**
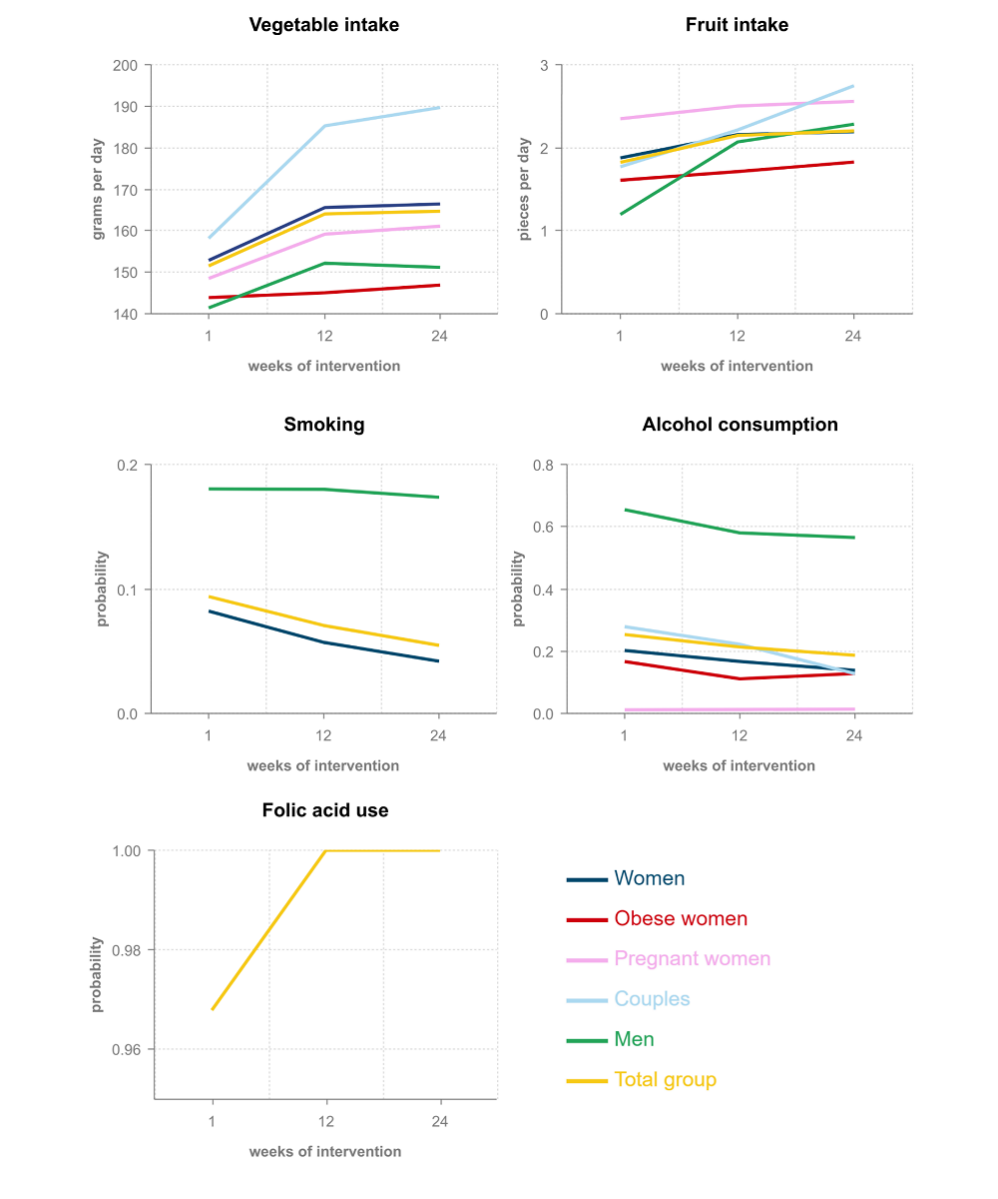
Improvement in lifestyle behaviors during online coaching in the total group and multiple subgroups.

Figure 2 depicts the improvement in lifestyle behaviors for the total group and the several subgroups during the 24 weeks of online coaching.

An increase in vegetable intake was observed after 24 weeks compared with baseline intake. This improvement was found for all subgroups; however, the strongest effect was found in women whose partners also participated in the blended periconception lifestyle care approach. These women showed a mean vegetable intake of 158 grams per day at baseline, and 185 grams per day after 12 weeks (*P*<.001) and 190 grams per day after 24 weeks of online coaching (*P*<.001). Women with obesity increased their vegetable intake from 144 grams per day at baseline to 145 grams per day after 12 weeks (*P*=.87) and 147 grams per day at the end of the online coaching program (*P*=.72).

Fruit intake increased in all subgroups and this improvement was the greatest in the group of male participants. This group showed almost doubling in fruit intake at the end of the online coaching program compared with baseline (1.4 pieces per day at baseline, 2.1 pieces per day at 12 weeks [*P*<.001], and 2.3 pieces per day at 24 weeks [*P*<.001]). Subgroup analysis in the group of women with obesity showed an increase in fruit intake from 1.6 pieces per day at baseline to 1.7 pieces per day at 12 weeks (*P*=.35) and 1.8 pieces per day at 24 weeks of online coaching (*P*=.05).

The probability of using tobacco decreased from 0.08 at baseline to 0.06 at 12 weeks (*P*=.004) and 0.04 at 24 weeks (*P*=.012) in the group of female participants. Tobacco use among male participants decreased from a probability of 0.18 at baseline to 0.17 at 24 weeks; however, this effect was not significant (*P*=.84). Additionally, the number of tobacco users was too low to perform further analyses in subgroups.

The probability of consuming alcohol decreased in all subgroups at the end of the coaching program compared with baseline. A significant effect was shown in the total study population and in the subgroup of women, with a decrease in probability from 0.25 to 0.19 (*P*<.001) and from 0.20 to 0.14 (*P*<.001), respectively. Alcohol consumption among male participants decreased from a probability of 0.95 to 0.82; however, this effect was not significant (*P*=.08).

The probability of having adequate folic acid supplementation increased from 0.97 at baseline to 1 after 12 weeks (*P*<.001). This effect was sustained after 24 weeks of online coaching (*P*<.001).

## Discussion

### Main Findings

This study confirms previous research reporting a high prevalence of inadequate lifestyle behaviors among women and men in the preconception period as well as during pregnancy [18]. Moreover, findings of this study suggest that a blended lifestyle care approach is an effective method to enhance a healthy lifestyle for couples who are either trying to conceive or who are pregnant. All targeted lifestyle components improved after the 24-week intervention. In particular, vegetable and fruit intake increased evidently among all study participants.

### Obesity

The results from women with obesity were less convincing. Vegetable and fruit intake in this group increased only marginally. However, this finding is in contrast to previous results in the study by van Dijk et al that showed positive effects of the Smarter Pregnancy program on lifestyle behaviors in the group of overweight and obese women [18]. In this study, subgroup analyses of lifestyle behaviors at baseline between women with obesity and the rest of the study population (including overweight women) showed no differences. Traditionally, obesity was thought to be a consequence of an individual’s poor lifestyle choices resulting in excess energy balance. Recently, the complexity of factors contributing to excess energy balance and weight gain have become more clear, and the following seven factors highly connected to the development of obesity have been identified: individual physiology, social psychology, individual psychology, individual physical activity, physical activity environment, food consumption, and food production [26]. Therefore, an intervention targeting unhealthy lifestyle components among women with obesity should not only focus on food consumption, but also address multiple factors in an integrated manner, including individualized psychological support.

### The Black Box of eHealth

The online coaching program Smarter Pregnancy has been proven to be effective for improving lifestyle behaviors [18,20,27]. However, the mechanism by which this intervention affects lifestyle behaviors and the elements that contribute the most are unclear and represent a “black box.” Opening this black box is even more relevant for the current blended care approach. Determining the core intervention principles, optimal frequency, and proportion between face-to-face counseling and eHealth could create an even more effective and efficient intervention.

### Strengths and Limitations

This is the first study to develop and evaluate a blended personalized lifestyle care approach for the periconception period, designed for both women and their partners. We included a high number of participants (n=511) and achieved a high intervention compliance rate of 78.9%. All lifestyle components improved after the 24-week intervention. However, the clinical relevance of increasing vegetable intake with 20 grams per day and fruit intake with 1 piece per day should be explored further, since no research has been performed to substantiate this effect. Extending follow-up and including data on sustainment of improved parental behavior and on maternal and neonatal pregnancy outcomes could provide insights on the clinical relevance of our findings.

The relatively low number of male participants may reflect their low level of engagement in a healthy lifestyle program. It might even demonstrate the low male involvement in pregnancy itself. However, it could also reflect the low extent to which women actively involve their partners in pregnancy. Future interventions should focus on raising awareness among expecting fathers of their role in reproductive health and pregnancy [28]. Considering that women who participated with their partners showed the strongest improvements in all lifestyle components, increasing the number of men participating in the approach might improve intervention effects. The current blended care approach could be extended with one face-to-face counseling focused on the partner. Another option that would be easy to realize and implement is the development of an informative animation video aimed at men that underlines the shared responsibility and opportunities to promote reproductive health. The reasons for the high effective rates among couples could include mutual support, eating warm meals together, and inspiring one another.

### Comparison With Prior Work and Implications for Future Research

A randomized controlled trial (RCT) is often considered to provide the most reliable evidence on the effectiveness of interventions. From this point of view, the absence of a control group (receiving only eHealth) is a possible limitation of this study. However, comparisons with previous research substantiate our findings. The study by Hammiche et al provided couples with tailored face-to-face preconception dietary and lifestyle counseling [17] and reported a compliance rate of only 26%. The survey by van Dijk et al included an even larger number of participants in the preconception or pregnancy period who received only the eHealth intervention Smarter Pregnancy [18]. This intervention study reported a compliance rate of 64.9%. The difference in compliance may be explained by the combination of face-to-face counseling with eHealth, resulting in higher participant engagement. Comparisons of effectiveness between the blended care approach and the survey with only the eHealth intervention should be made with caution, since information on possible confounding factors is missing. Furthermore, in recent years, there has been increasing discussion about the limitation of traditional RCT methodologies for the evaluation of eHealth interventions [29]. Locking down these interventions results in inclusion of possible defects and eliminates the opportunities for quality improvement and adaptation to the changing technological environment, often leading to validation of tools that are outdated by the time trial results are published. Therefore, a design that evaluates the intervention principles (rather than a specific locked-down version of the intervention) is more appropriate. Using the “Trial of Intervention Principle” evaluation method, the design allows for ongoing quality improvement modifications to the behavioral intervention technology based on the core intervention principles, and it continuously improves functionality and maintains technological currency [30].

Further research could be performed on how to continue the steep increase in vegetable and fruit intake after 12 weeks, since our study showed an almost flat curve after this intervention period. The plateau in vegetable and fruit intake might be explained by intervention elements applied in the Smarter Pregnancy program. This intervention particularly focused on increasing external motivation and providing education. It might be effective to add elements that support behavior change in the long term, such as person action plans, goal setting, and maintenance plans [31].

The next step is to further disseminate the approach. Additionally, follow-up of our study participants and their pregnancy outcomes is needed to substantiate the clinical relevance of the results of this study.

## Abbreviations

BMI: body mass index

RCT: randomized controlled trial
